# DIetary ASSessment (DIASS) Study: Design of an Evaluation Study to Assess Validity, Usability and Perceived Burden of an Innovative Dietary Assessment Methodology

**DOI:** 10.3390/nu14061156

**Published:** 2022-03-09

**Authors:** Desiree A. Lucassen, Elske M. Brouwer-Brolsma, Anne I. Slotegraaf, Esther Kok, Edith J. M. Feskens

**Affiliations:** Department of Agrotechnology and Food Sciences, Division of Human Nutrition and Health, Wageningen University and Research, 6700 AA Wageningen, The Netherlands; elske.brouwer-brolsma@wur.nl (E.M.B.-B.); anne.slotegraaf@wur.nl (A.I.S.); esther.kok@wur.nl (E.K.); edith.feskens@wur.nl (E.J.M.F.)

**Keywords:** dietary assessment, technology, EMA, recall, FFQ, diet quality, validation, nutritional biomarkers

## Abstract

During recent years, the integration of technology has substantially improved self-reported dietary assessment methods, such as food frequency questionnaires (FFQ), food records, and 24-h recalls. To further reduce measurement error, additional innovations are urgently needed. Memory-related measurement error is one of the aspects that warrants attention, which is where new smartphone technologies and ecological momentary assessment (EMA) approaches provide a unique opportunity. In this article, we describe the DIASS study, which was designed to evaluate an innovative 2-h recall (2hR) smartphone-based methodology, against traditional 24-h recalls, FFQ, and biomarkers, to assess both actual and habitual dietary intake. It is hypothesized that a 2-h reporting window decreases reliance on memory and reporting burden, and increases data accuracy. We included 215 men (28%) and women (72%), with a mean ± SD age of 39 ± 19 years and a mean ± SD BMI of 23.8 ± 4.0. Most participants were highly educated (58%). Response rates for the various dietary assessment methods were >90%. Besides the evaluation of the accuracy, usability, and perceived burden of the 2hR methodology, the study set-up also allows for (further) evaluation of the other administrated dietary assessment tools.

## 1. Introduction

Accurate dietary assessment is one of the essential aspects of nutrition and health (behavior) research, where 24-h recalls (24hRs), food frequency questionnaires (FFQs), and food records are currently the most commonly-used dietary assessment methods [1,2,3,4]. However, these methods have a range of drawbacks [2,5]. FFQs and 24hRs are retrospective and, thus, memory dependent, which makes recall bias [6,7] and misreporting nearly inevitable [2,8,9]. A food record is not memory dependent, but its prospective nature may introduce reactivity bias, due to, for instance, social desirability or to ease the recording task [2,7,9]. Finally, all these methods appear to heavily burden both the participant and the researcher [5,7,10].

Accordingly, there is a growing interest in more technology-based dietary assessment methods, which have the potential to improve accuracy and reduce the burden on both participant and researcher [3,11,12]. Numerous valuable computer- and web-based tools, mostly based on 24hRs and FFQs, have been developed during the past decade [3,10,12,13]. More recently, various smartphone applications (i.e., apps) have been developed to collect dietary intake data via digital food records [3,7,12]. Nevertheless, due to their self-report nature, prospective apps are still prone to reactivity bias and still highly intrusive [5,7,10], as illustrated by the time needed to register food intake [3].

To the best of our knowledge, no (validated) recall-based dietary assessment apps exist at present [12]. Although an innovative retrospective app still relies on the participants’ memory [2,4,7], apps have the major advantage of enabling (near) real-time collection of dietary intake data [3,7,14,15]. In behavioral and social sciences, this is referred to as ecological momentary assessment (EMA); repeated real-time assessment of individual’s behavior in their natural environment, where the ecological aspect focuses on the individual’s ‘real-world’ and the momentary aspect on the individual’s current or very recent state [15]. EMA opens the possibility of deviating from traditional dietary assessment methods and exploring new data collection efforts. More specifically, it offers the opportunity of deviating from the traditional 24hRs, to shorter recall periods (e.g., 2-h, 4-h, 8-h), which reduces the reliance on a participants’ memory, takes less time to complete, and, thus, should have a lower burden for the respondent.

Therefore, we developed an innovative smartphone-based dietary assessment app [16] that can serve to collect dietary intake data in a faster, more flexible, and more reliable manner than the traditional methods. In order to facilitate tailored use, and depending on the purpose of dietary intake collection, the app can be used in the format of a food record or recall. The recall-module is also flexible, in terms of the reporting window; enabling 1-h recalls up to 24hRs. Within the current study, we explored the use of an innovative 2-h recall (2hR) methodology for (near) real-time data collection. A 2-h reporting period minimizes the reliance on memory compared to the traditional 24hRs. The 2-h reporting window was selected over a 1-h reporting window to avoid ‘I did not consume anything’ responses; e.g., overburdening of the participant. A longer reporting window of, e.g., 3-h or 4-h, was considered, but repudiated owing to a higher memory-related and reporting burden. Therefore, we felt that a 2-h reporting window would result in the lowest participant (perceived) burden relative to the report of a limited number items at once, while limiting memory-related bias. The 2hR methodology is flexible and can be used to assess actual food intake, by sending consecutive 2hRs on one or more full-days or dayparts, depending on the research question. The 2hRs can also be used to assess habitual intake, by sending random 2hRs over a longer period of time. However, it needs to be stressed that an adequate sampling scheme is crucial here, i.e., ensuring equal coverage of all eating occasions, allowing the assessment of habitual intake.

Although the 2hR methodology sounds promising, its validation against established methods is imperative, to judge its actual value. The DIASS study was designed to evaluate the accuracy of the smartphone-based 2hR methodology, to assess both actual and habitual intake of food groups, energy, and nutrients compared to established methods and independent biological markers. Secondary aims included the evaluation of the usability, perceived burden, and compliance of the 2hR method compared to established methods. Additionally, the DIASS study allows further evaluation of the other administrated dietary assessment tools. With this article, we aim to provide an overview of the (1) study design of the DIASS study, and (2) baseline characteristics of the study population, as a reference for future evaluation studies that will be performed using these data.

## 2. Materials and Methods

### 2.1. Design

The DIASS study had a cross-over design (12-weeks), with two experimental conditions; i.e., measuring actual intake and habitual intake. Dietary intake was assessed by means of the new 2hR app, as well as various established methods. In addition, information on demographics (e.g., educational level, occupation) was collected by means of an online questionnaire derived from the NQplus questionnaire [17]. Additional questions were included regarding the participant’s weight stability and sleep pattern. Height and weight were measured on-site. During the study, activity trackers were used to assess physical activity levels. In the final study week participants were invited to complete an evaluation questionnaire regarding the various dietary assessment methods.

**Figure 1:**
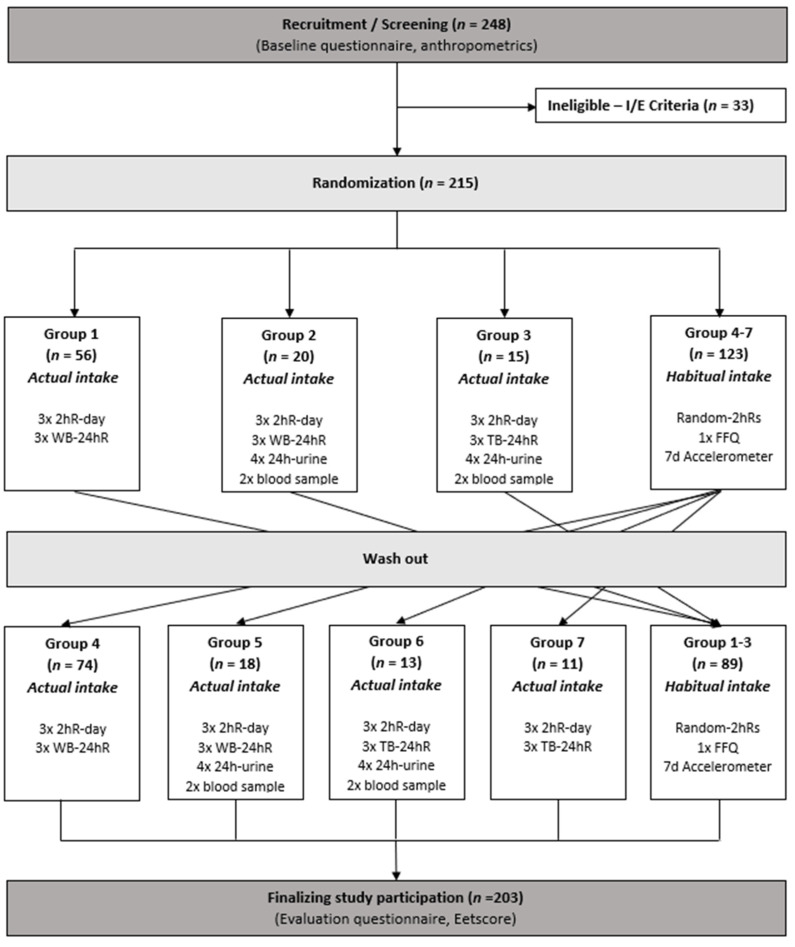
Flowchart study design. 2hR-day: Full day of consecutive 2-h recalls; WB-24hR: web-based 24-h recall; 24-h urine: 24-h urine collection; TB-24hR: telephone-based 24-h recalls; Random-2hRs: Randomly distributed 2-h recalls; FFQ: food frequency questionnaire; Eetscore: Web-based screener for diet quality.

Originally, the 215 participants were randomly allocated into six groups (Figure 1). Groups differed in terms of the additional methods used to assess actual intake; groups did not differ in terms of the methods used to assess habitual intake. Therefore, the habitual groups in Figure 1 are combined. Unfortunately, due to the COVID19 pandemic, the urine and blood collections of 31 participants (groups 2, 3, 5, 6), scheduled from March 2020 onwards, were cancelled. Consequently, these participants were relocated to matching groups without urine and blood collections (groups 4, 6, and newly formed 7).

After randomization in week 1, each participant completed two study periods of four weeks each (i.e., week 2–5 and week 8–11), during which, either actual or habitual intake was assessed, in random order. To minimize the participant burden, and as such optimize compliance, the two study periods were separated by a wash-out period of two weeks (i.e., week 6–7). Additionally, overall diet quality was assessed in week 12.

The DIASS study was approved by the ethics committee of Wageningen University and Research (WUR) (ABR No.: NL69065.081.19) and conducted according to the guidelines laid down in the Declaration of Helsinki.

### 2.2. Participants

Recruitment took place between June 2019 and May 2020, and aimed to include 220 Dutch adults (men and women) aged 18–70 years. Eventually, 215 men and women were included in the study. Participants were recruited via the research volunteer database of the division of Human Nutrition and Health of WUR, social media accounts of the division (i.e., Twitter, Facebook), and through flyers and posters. Participants had to be Dutch speaking, not visually impaired, in possession of a smartphone with internet plan, metabolically stable (i.e., gained or lost ≤3 kg in the past 3 months), willing to maintain current dietary habits for the duration of the study, not participating in another dietary intervention study, not an employee of the division, and not having any formal training in the field of nutrition. Written informed consent was obtained from all participants prior to participation.

### 2.3. Dietary Intake Assessment

Actual food intake was assessed on random non-consecutive days over one of the four-week study periods. Within this period, participants were invited to complete three 2hR-days and three 24hRs. In addition, a random subsample of 69 participants also provided four 24-h urine samples and two fasting blood samples. The urine collections were intentionally coupled to the recall days (i.e., 2× to 2hR-day and 2× to 24hR-day). Blood sampling occurred following two of the urine collections while the participants were at the study center to hand in their urine containers. During the other four-week study period, habitual food intake was assessed by random 2hRs. The same number of 2hRs was used as for the 2hR-days. However, to assess habitual intake, the 2hRs were randomly distributed over the four-week period (i.e., 3× each time slot). In case of non-response, the 2hR was automatically rescheduled on the same time on another day. In addition, at the end of the study period, participants were invited to complete a FFQ. In the final study week, participants were invited to complete an additional short FFQ, to assess overall diet quality.

#### 2.3.1. 2hRs

The 2hRs were sent via Traqq^®^, a dietary assessment app developed by WUR [16]. By clicking on the notification/opening Traqq^®^, participants were able to report their food intake of the previous 2 h. The food intake report screen is supported by an extensive food list, based on the Dutch Food Composition Database [18]. Following the reporting of the food, participants are requested to enter the consumed amount and eating occasion (i.e., breakfast, lunch, dinner, snack). Amount was reported in household measures (e.g., cups, spoons), standard portion size (e.g., small, large) [19] or amount in gram. Participants could also report all ingredients of a recipe and the amount consumed (i.e., yield and retention factors are automatically taken into account) under the ‘My Dishes’ feature. In addition, this function could also be used to create frequently consumed food combinations (e.g., daily breakfast) to simplify the reporting of these items. When participants did not consume anything in the specified time window they could simply press the ‘I did not eat or drink anything’ button [16].

**Figure 2:**
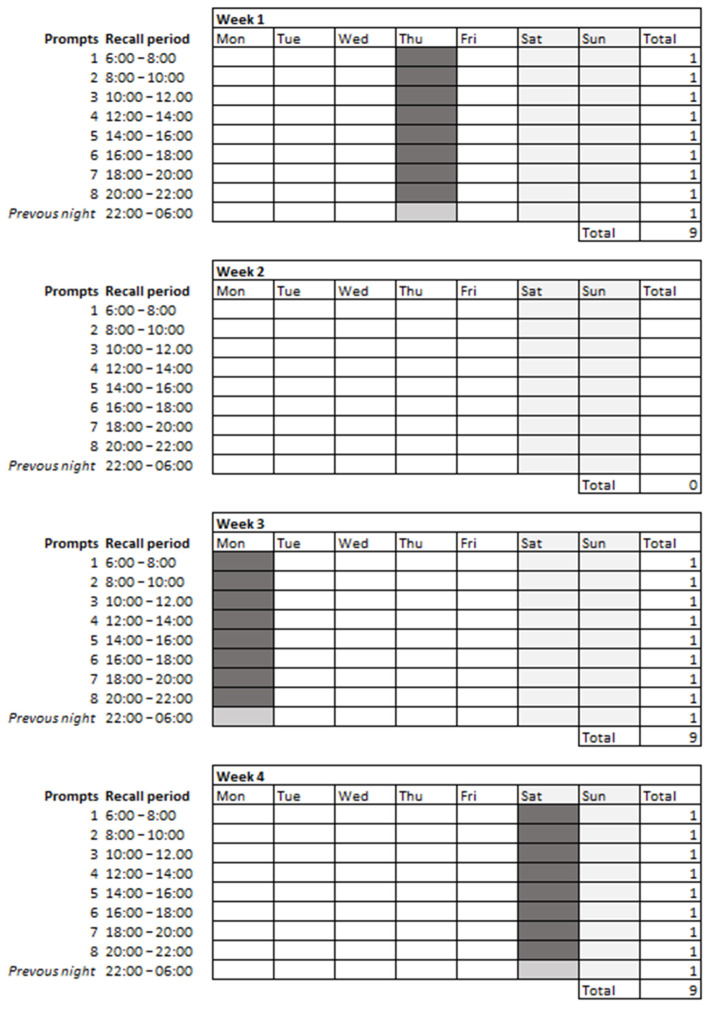
Example actual intake sampling scheme (i.e., three 2hR-days).

Actual intake was measured on thee random days by means of multiple consecutive 2hRs per day (Figure 2). A subsample also collected 24-h urine samples. Therefore, for this group, two of the 2hR-days were first randomly scheduled and then communicated to the participant to ensure 24-h urine collection on the recall day. The remaining 2hR-day was unannounced. On the recall days, participants received an invitation every 2 h to report their food intake during the previous 2 h (Figure 2). To ensure complete data collection, participants also received an additional prompt the following morning, i.e., to report any remaining food intake from the previous night. To stimulate a quick response [20], participants were informed that a 2hR closed after 60 min. However, in reality, the 2hRs remained open until the end of the day and then closed automatically. Participants were allowed to miss one invitation on a 2hR-day. In case of >1 missed 2hRs, the sampling was seen as incomplete and a new recall day was scheduled. On average, participants received eight consecutive 2hR invitations on a recall day. The 2hR-day sampling scheme was individualized according to the participant’s sleep pattern (i.e., inquired though the baseline questionnaire).

**Figure 3:**
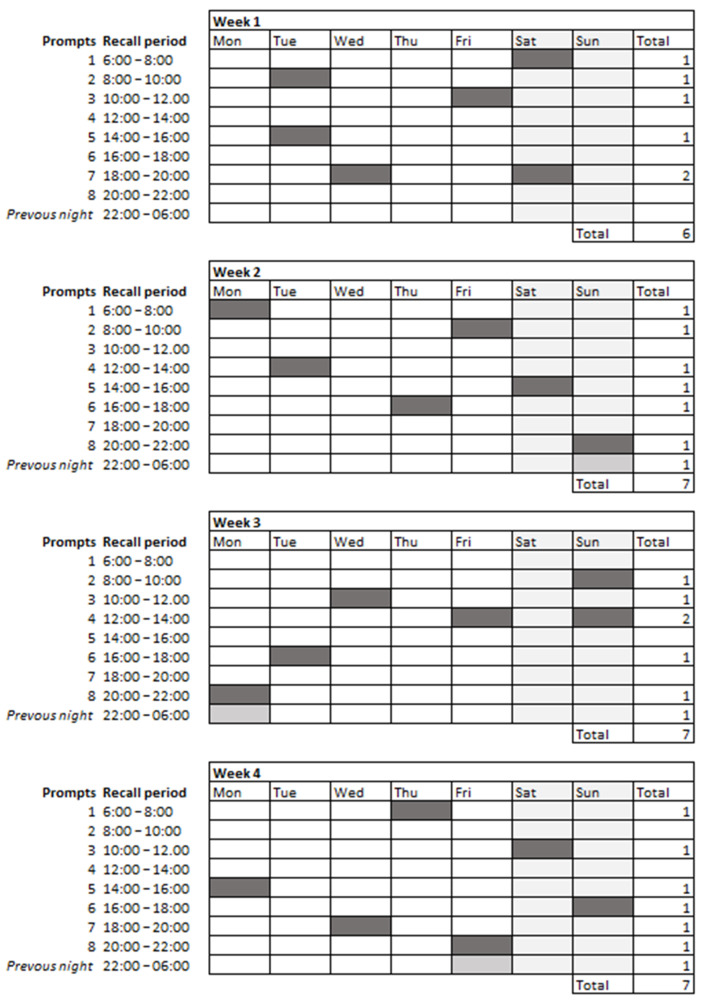
Example habitual intake sampling scheme (i.e., three times each 2hR-slot).

Habitual intake was measured by multiple random 2hRs. Participants received an invitation on random days and times to report their food intake during the previous 2 h (Figure 3). The same timeslots were used as for the actual intake measurement, but now, randomly divided over the four-week period instead of combined on full days. The 2hRs were restricted to a maximum of two per day to limit the number of recordings on one day. However, the additional question regarding the previous night remained linked to the final evening 2hR. Participants had a 60-min response deadline. In case of non-response, the 2hR closed, and a new invitation was automatically rescheduled for the same time on a different day.

#### 2.3.2. Web-Based 24 h

A total of 168 participants were invited to complete thee random web-based 24hRs with Compl-eat™. A subsample of 38 of these participants also collected 24-h urine samples. Consequently, for this group, two of the 24hRs were randomly scheduled and then communicated to the participant to ensure 24-h urine collection on the recall day. The remaining 24hR was unannounced. Compl-eat™ is a validated self-administered web-based dietary 24hR-tool developed by WUR and is based on the automated five-step multiple-pass method [21]. In this method, participants first fill in a quick list of consumed foods and then in the next steps provide detailed information about eating occasion, type of foods, and consumed quantities [22]. The method of reporting intake in Compl-eat™ is similar to the reporting method in Traqq^®^. Items can be searched for in the food list, and consumed amounts can be reported in similar household measures, standard portion sizes, or in grams [19,22]. Compl-eat™ contains a recipe module similar to the ‘My Dishes’ function of Traqq^®^. Moreover, participants were able to make notes for clarifications. Invites for the web-based 24hRs were sent via email at 06:00 in the morning of the recall day. The questionnaire was accessible until midnight that same day. In case of non-response, a new 24hR was randomly scheduled.

#### 2.3.3. Telephone-Based 24 h

A subsample of 39 participants were asked to complete interviewer-administered telephone-based 24hRs instead of web-based 24hRs. A subsample of 28 of these participants also collected 24-h urine samples. Therefore, two of the 24hRs were randomly scheduled and then communicated to the participant to ensure 24-h urine collection on the recall day. The final 24hR remained unannounced. If needed, the dieticians made multiple attempts to reach the participant by phone on a recall day. In case of non-response, a new 24hR was randomly scheduled.

Since the validation of Compl-eat™, improvements have been made to the tool that still need to be validated. Therefore, the telephone-based 24hRs were used to ensure the accuracy of Compl-eat™. The telephone-based 24hRs were conducted by trained dieticians using a standardized protocol and the five-step multiple-pass approach [22]. However, to minimize reactivity bias, participants were not informed that the interviews were conducted by dieticians, in attempt to minimize socially desirable answers.

#### 2.3.4. Computation of Dietary Recall Data

Data from both the 2hRs and the 24hRs were entered in the computation module of Compl-eat™ [21]. Total intakes of energy, macro-, and micronutrients, and food group intakes (g/d) were calculated using the Dutch Food Composition Database 2016 [18]. Dietary intake data were thoroughly checked by trained dieticians, according to a standardized protocol. The dieticians checked the data for completeness and unusual amounts. Errors were corrected according to a standardized approach, using standard portion sizes and recipes (e.g., 35 slices of bread was corrected to 1 slice of 35 g). Participants were not contacted in case of discrepancies.

#### 2.3.5. FFQ

All participants were asked to complete a validated 183-item semi-quantitative FFQ, with a reference period of four weeks [23]. This extensive FFQ was administered online with the self-administered Dutch FFQ-tool™ [24]. Participants indicated the frequency of consumed food items by selecting answers ranging from ‘not consumed’ to ‘7 days per week’. In addition, portion sizes were estimated using natural portions and commonly used household measures. Energy and nutrient contents of foods were based on the Dutch Food Composition Database 2010 [25] and multiplied by the portion size and frequency of consumption to calculate mean daily intake of energy, macro-, and micronutrients. In addition, average daily intake (in grams) of food items were calculated by multiplying frequency of consumption by portion size. Trained dieticians conducted multiple quality checks to safeguard the quality of the data.

#### 2.3.6. Diet Quality

All participants were asked to complete the Eetscore™ in the final study week. The Eetscore™ is a self-administered web-based screener for diet quality [26]. It consists of a 55-item FFQ and is scored with the Dutch Healthy Diet 2015-index to evaluate adherence to the Dutch food-based dietary guidelines [27]. This short FFQ was administered online with the Dutch Eetscore-tool™. Participants indicated frequency of consumed food items by selecting answers ranging from ‘never’ to ‘every day’ for regularly consumed foods and from ‘not this month’ to ‘four times a month’ for episodically consumed foods (e.g., legumes). Portion sizes were estimated using natural portions and commonly used household measures. Average daily intake of food items were calculated by multiplying frequency of consumption by portion size in grams. Sodium content of food items were based on the Dutch Food Composition Database 2010 [25] and multiplied by the portion size and frequency consumption.

### 2.4. Urine Collection

A total of 66 participants provided four 24-h urine samples during the ‘actual intake’ period. Two of these samples were linked to 2hR-days and the other two to 24hRs. The participants were instructed on 24-h urine sampling according to a standardized protocol and were provided with three-liter containers containing the preservative lithium dihydrogenphosphate (25 g). Participants also received three 100 mg para-aminobenzoic (PABA) tablets (KAL Vitamins, Salt Lake City, UT, USA), and were instructed to ingest one PABA tablet with each main meal. The 24-h urine collection started with the second voiding after waking up and was completed with the first voiding after waking up the next day. Participants were instructed to record the beginning and end times of the 24-h urine collection, the time of ingesting the PABA tablets, and any possible deviations from the protocol (e.g., missing urine). Urine samples were handed in at the study center where they were mixed, weighed, aliquoted, and stored at −80 °C until further analysis.

PABA was provided to check for completeness of the 24-h urine samples. Research has shown that providing PABA is recommended, but that it does not necessarily have to be analyzed [28]; often creating a feeling of being observed is enough. Moreover, when participants are willing to commit to four 24-h-urine collections, two blood samplings, and four extra visits to the study center, compliance often follows [29]. However, the 24-h urine collections were determined as valid if they met all of the following criteria: (1) collection time of 22–26 h, (2) sample volume ≥500 mL, (3) no more than 1 reported missed void, (4) estimated missed volume ≤5% of the total volume, and (5) creatinine levels of >10 mg/kg for women and >15 mg/kg for men [28]. Urinary creatinine was measured at 520 nm on the Synchon LX20 by the modified Jaffé procedure using a commercial kit.

The 24-h urine samples were assessed on nitrogen, potassium, and sodium content, which were used to estimate absolute intakes of protein, potassium, and sodium, respectively [30,31]. Urinary 24h-nitrogen (N) excretion was determined with the Kjeldahl technique (Foss KjeltecTM 2300 analyzer; Foss Analytical). Urinary protein content was calculated with the following formula: 6·25 × (urinary N/0·81), accounting for an assumed 19% of fecal and skin losses [28,32]. Additionally, urinary potassium (K) concentration measurements were performed with an ion-selective electrode on a Roche 917 analyzer. The 24-h K-excretion was calculated by multiplying the total weight of the 24-h urine sample by the K-concentration. Following, this was divided by 0.77, assuming an urinary excretion of 77% [28]. Finally, urinary sodium (Na) was calculated the same way as urinary K. However, an urinary excretion of 86% was assumed [28]. The remaining 24-h urine samples were stored at −80 °C for additional analyzes.

### 2.5. Blood Collection

The 66 participants that provided 24-h urine samples also provided two fasting blood samples. Following a 10-h overnight fast, these participants underwent a venipuncture at the study center. The venipunctures were conducted by experienced staff members and scheduled on days that participants were already at the study center to hand in their 24-h urine samples, sparing them extra visits. Biochemical analyses were performed either on a Dimension Vista 1500 automated analyzer (Siemens, Erlangen, Germany) or a Roche Modular P800 chemistry analyzer (Roche Diagnostics, Indianapolis, IN, USA). The blood samples were used to assess carotenoid, folate and *n*-3 fatty acid concentrations, to estimate habitual intake of fruit and vegetables, folate, and fish, respectively [31,33,34]. The remaining plasma and serum samples were stored at −80 °C for additional analyzes.

### 2.6. Anthropometrics

Anthropometrics were conducted by trained staff, according to a standardized protocol in study week 1. Height was measured without shoes, using a stadiometer (SECA 213; SECO Corp., Hamburg, Germany) to the nearest 0.1 cm. Weight was measured without shoes, heavy clothing, and with empty pockets on a digital scale (SECA 877; SECA Corp., Hamburg, Germany) to the nearest 0.1 kg. Weight measurements were repeated in study weeks 7 and 12, to determine weight stability.

### 2.7. Total Energy Expenditure

Total energy expenditure (TEE) is estimated by calculating basal metabolic rate (BMR) and assessing physical activity level (PAL). BMR was calculated using the Harris and Benedict equation [35]. In addition, PAL was assessed over a 7-day period, in which the participant wore the ActiGraph wGT3X-BT accelerometer (ActiGraph LLC, Pensacola, FL, USA), except when showering, bathing, swimming, or involved in contact sports [36]. Accelerometers have been reported to be objective, practical, non-invasive, accurate, and reliable tools to assess physical (in)activity [36,37].

The raw accelerometer data was downloaded from the ActiGraph devices and thoroughly checked by an experienced data scientist. Participants were included if the devices collected accelerometer data of 7 consecutive days. Next, the data were imported into ActiLife version 6.13.4 (ActiGraph LLC, Pensacola, FL, USA), and participant’s percentage of time spent in sedentary, light, moderate, vigorous, and very vigorous activity using the Troiano algorithm [38]. The daytime activity percentages were then extracted from ActiLife and multiplied with the corresponding PALs using Python version 3.7 (Python Software Foundation, Wilmington, DE, USA), according to the guidelines set by the WHO. The WHO guidelines describe a mean PAL, based on factorial calculations of the time spent on activities during the day and the energy cost of those activities (i.e., sedentary: 1.4, light activity: 1.55, moderate: 1.7, vigorous: 1.8, very vigorous: 2.2) [39]. This process resulted in an individual PAL for each participant.

### 2.8. Demographics

The baseline questionnaire acquired general participant information (i.e., age, gender, educational level, daytime activities, sleeping pattern, intention to maintain current body weight). This questionnaire was derived from the NQplus study, a large cohort study in the Netherlands [17]. Additional questions were included to assess a participant’s sleep pattern in order to personalize the 2hR sampling times.

### 2.9. Evaluation Questionnaire

In the final study week, participants completed an evaluation questionnaire on their experiences using the app and various commonly-used conventional dietary assessment methods. The evaluation questionnaire was based on previous studies and assessed aspects such as ease of use, convenience, perceived reporting burden, perceived accuracy, likelihood of future use, and overall experience [40,41]. Responses were based on a 5-point Likert scale (i.e., strongly agree, agree, neutral, disagree, strongly disagree), or participants could indicate what dietary assessment method matched best with a specific statement. In addition, the participants were asked to complete the system usability scale (SUS) for Traqq® [42]. This is an 10-item questionnaire with a 5-point Likert scale ranging from 1 (strongly disagree) to 5 (strongly agree). The SUS has been used in previous studies to assess the usability of dietary assessment apps [43,44].

## 3. Statistical Analyses

Baseline characteristics are presented as means with standard deviations (SD) and frequencies (*n*) with percentages (%). The SUS score was calculated with a predefined formula (range 0–100). A SUS score of >68/100 indicates an above-average usability and a score of >80/100 indicates an excellent usability [42]. Data analyses were performed using SPSS Statistics version 25.0 (SPSS Inc., Chicago, IL, USA).

## 4. Baseline Characteristics

In total, 215 men (28%) and women (62%) were included in the DIASS study (Table 1). The participants had a mean ± SD age of 39 ± 19 years and a mean ± SD BMI of 23.8 ± 4.0 kg/m^2^. According to the accelerometer data the participants had a sedentary lifestyle (mean ± SD PAL of 1.46 ± 0.02). The majority of the participant were either <25 years (42%) or ≥50 years (38%), most of the participants were highly educated (58%), the majority of the participants were either married/registered partners (32%) or single (42%), over half of the participants (52%) had a paid job, and the majority of the participants did not follow a diet regimen (71%). Overall, response rates for the dietary assessment methods were high (>90%). The completeness of the urine collections was 86%, and 100% of the fasting blood samples were collected. Finally, mean SUS score of the app was 72 ± 14.

## 5. Discussion

We have described the design of the DIASS study, which aimed to evaluate a newly developed smartphone-based dietary assessment methodology against established methods and objective markers. A total of 215 men and women were included (18–70 years); mainly women and highly educated. The overall response rates were high with >90% for the dietary assessment approaches and >86% for the collection of biological samples.

Various dietary assessment apps have been developed for research purposes, all based on the food record approach. Similarly to Traqq^®^, most of these apps rely on text entry for food identification and quantification; respondents select consumed foods from a fixed food list and quantify amounts by weights or household measures [12,41,45,46,47,48]. In contrast, some of the available apps rely on digital images for food identification and quantification, i.e., respondents take a before and after picture of each meal. Although this approach seems promising these apps are not fully automated, i.e., require some form of manual image review by user and/or researcher [12,43,44,49,50,51]. The text- and image-based dietary assessment apps all rely on national databases as the source of food composition data; thus, ensuring the quality of the nutrition calculations. Validation studies of these apps also show good agreement between the apps and the reference methods [12,43,48]. These comparisons are mostly made against established methods (e.g., 24hRs, weighed food records). A limited number of validation studies also included objective measures for total energy expenditure (TEE) from doubly-labelled water or accelerometers [49,50,52]. However, objective measures for nutrient intakes (i.e., biomarkers) are generally lacking [12,43,48]. Despite their limited availability [30,31], biomarkers are more sensitive for quantifying the magnitude and direction of potential measurement errors than traditional self-report dietary assessment methods [53]. Therefore, an important strength of this evaluation study was the collection of biological samples, which offered the opportunity to conduct established urine- and blood-based nutrient biomarker assessments, including nitrogen, potassium, sodium, folate, carotenoids and EPA/DHA, as well as more innovative food metabolomics [54]. Moreover, similarly to a few of the validated apps, we used accelerometers to obtain an objective measure for TEE.

The SUS score of 72 indicates that the app Traqq® has a good usability. This SUS rating was slightly lower compared to our prior usability test (mean SUS of 79) [16]. However, it should be noted that participants in our previous study only used the app for approximately one hour while performing specific tasks in a controlled environment, while during the DIASS study, participants used the app for two periods of four weeks in their day-to-day lives. Obviously, more issues occur with prolonged use (e.g., more missing food items or connectivity issues). 

The DIASS study may be considered limited by the fact that about 70% of the participants were highly educated women, which may limit the generalizability of the results. However, this validation study was designed to assess the accuracy of the 2hR-based dietary intake estimates. Therefore, the lack of generalizability may not be as much of a limitation as it would have been in research into diet–disease relationships. However, acceptability and usability levels might be lower for individuals with a lower educational level [55,56]. Therefore, for usage in other study populations, it will be important to perform additional evaluations, and alterations to the method might be required.

Unfortunately, the COVID-19 pandemic forced us to cancel the final blood and urine collections, to safeguard the participants’ health. This decreased the number of participants that collected independent biomarkers, from 100 to 69 participants. However, the data from the remaining participants in this subsample still provide valuable insights into the relation between true and assessed intake. Although this last sample of participants were in lockdown for the entire study duration, the self-reported dietary assessment and additional measures could proceed as planned.

In conclusion, the most important feature of the DIASS study is its elaborate study design. The results consist of actual dietary intake data obtained by multiple 2hR-days, multiple web-based or telephone-based 24hRs, and biochemical markers. This allows validation of actual intake assessment with the 2hR-days against both established methods and independent urinary biomarkers. Moreover, the same data can also be used to validate the most recent version of Compl-eat™. In contrast, the habitual dietary intake data obtained by multiple random 2hRs, FFQ, and, again, biochemical markers allows for validation of habitual intake assessment. This data also allow for further validation of the FFQ. Finally, the results of the Eetscore™ can be validated against the results of the FFQ and the 24hRs, as the diet quality score used in the Eetscore™ can also be calculated from these more extensive approaches. Additionally, the collected data can also be used to evaluate all administered methods, in terms of usability, response time, and perceived burden. As such, we believe that the DIASS study offers a unique opportunity for extensive evaluation of a variety of dietary assessment methods and contributes to the further improvements of these methods.

## Figures and Tables

**Table 1:** Baseline characteristics of the DIASS participants, including collected dietary intake data.

	N	Total	Men	Women
Men (*n*, %)	215	60 (28)	60 (100)	155 (0)
Mean age, years (SD)	215	39 (19)	45 (19)	37 (18)
Age category (*n*, %)	215			
<25 years		90 (42)	19 (32)	71 (46)
25–50 years		43 (20)	10 (16)	33 (21)
≥50 years		82 (38)	31 (52)	51 (33)
Mean BMI, kg/m^2^ (SD)	215	23.8 (4.0)	25.0 (4.3)	23.4 (3.8)
BMI category (*n*, %)	215			
<18.5 kg/m^2^		7 (3)	1 (1)	6 (4)
18.5–25 kg/m^2^		148 (69)	34 (57)	114 (73)
≥25 kg/m^2^		60 (28)	25 (42)	35 (23)
Mean BMR, kcal/day (SD)	215	1545 (211)	1799 (174)	1446 (122)
Mean PAL	203	1.46 (0.02)	1.46 (0.02)	1.46 (0.01)
Educational level (*n*, %)	215			
Low		5 (2)	0 (0)	5 (3)
Intermediate		85 (40)	26 (43)	59 (38)
High		125 (58)	34 (57)	91 (59)
Marital status (*n*, %)	215			
Married/registered partnership		69 (32)	25 (42)	44 (28)
Cohabiting		25 (12)	8 (13)	17 (11)
Serious relationship, not cohabiting		20 (9)	6 (10)	14 (9)
Single		90 (42)	17 (28)	73 (47)
Divorced		7 (3)	3 (5)	4 (3)
Widowed		3 (1)	0 (0)	3 (2)
Other		1 (1)	1 (2)	0 (0)
Paid job currently (*n*, %)	215			
Yes		112 (52)	33 (55)	79 (51)
No		103 (48)	27 (45)	76 (49)
Diet regimen (*n*, %)	204			
Yes, always		35 (17)	4 (7)	31 (21)
Yes, sometimes		24 (12)	6 (11)	18 (12)
Never		145 (71)	45 (82)	100 (67)
Number of complete dietary data collections (*n*, %)				
2hR-day ^1^	214	591 (92)	158 (88)	433 (94)
WB-24hR	167	474 (90)	126 (88)	348 (91)
TB-24hR	39	117 (98)	33 (92)	84 (100)
Linked 24-h urine collections	66	238 (86)	73 (83)	165 (88)
Blood sample	66	138 (100)	44 (100)	94 (100)
Random 2hRs	212	4669 (96)	1322 (95)	3347 (96)
FFQ	212	204 (96)	55 (92)	149 (98)
Eetscore	203	192 (95)	54 (98)	138 (93)
Mean System Usability Score (SD)	190	72 (14)	73 (15)	72 (13)

^1^ No more than one 2hR missed per day.
